# Feather Corticosterone Measurements and Behavioral Observations in the Great White Pelican (*Pelecanus onocrotalus*) Living under Different Flight Restraint Conditions in German Zoos

**DOI:** 10.3390/ani11092522

**Published:** 2021-08-27

**Authors:** Gudrun Haase, Mechthild Wiegard, Christa Thöne-Reineke, Katrin Baumgartner, Lorenzo von Fersen, Hermann Will, Roswitha Merle, Manel Lopez-Bejar, Oriol Tallo-Parra, Annais Carbajal, Lukas Reese

**Affiliations:** 1 Animal Behavior and Laboratory Animal Science, Institute of Animal Welfare, Freie Universität Berlin, Königsweg 67, D-14163 Berlin, Germany; mechthild.wiegard@fu-berlin.de (M.W.); Christa.Thoene-Reineke@fu-berlin.de (C.T.-R.); 2 Vogelpark Marlow, Kölzower Chaussee 1, D-18337 Marlow, Germany; 3 Zoo Nuremberg, Am Tiergarten 30, D-90480 Nuremberg, Germany; Katrin.Baumgartner@stadt.nuernberg.de (K.B.); lorenzo@vonfersen.org (L.v.F.); Hermann.Will@stadt.nuernberg.de (H.W.); 4 Institute for Veterinary Epidemiology and Biostatistics, Freie Universität Berlin, Königsweg 67, D-14163 Berlin, Germany; Roswitha.Merle@fu-berlin.de; 5 Veterinary Faculty, Universitat Autònoma de Barcelona, Campus UAB, ESP 08193 Bellaterra, Spain; Manel.Lopez.Bejar@uab.cat (M.L.-B.); Oriol.Tallo@uab.cat (O.T.-P.); anais.carbajal@uab.cat (A.C.); 6 College of Veterinary Medicine, Western University of Health Sciences, Pomona, CA 91766, USA; 7 Zoologischer Stadtgarten Karlsruhe, Ettlinger Straße 6, D-76137 Karlsruhe, Germany; Lukas.reese@zoo.karlsruhe.de

**Keywords:** feather corticosterone, great white pelican, zoo animal welfare, pinioning, clipping feathers, behavior, deflighting

## Abstract

**Simple Summary:**

The welfare of zoo birds kept under flight restraint is a frequently discussed topic. Therefore, this study was conducted with one of the most regularly kept types of deflighted birds in German zoos, the great white pelican, to find scientific data regarding welfare assessments of deflighted birds. The detection of corticosterone in feathers (CORTf) as a stress indicator for birds is an almost completely noninvasive form of measurement meant to evaluate the effects of deflighting birds in zoos. Three groups of animals were compared: irreversibly deflighted pelicans that were pinioned or extirpated, reversibly deflighted individuals that were feather-clipped, and airworthy pelicans that were able to fly. Combining two independent research methods, behavioral observation and the measurement of CORTf levels of great white pelicans, we aimed to obtain an objective overview of whether deflighted birds showed differences in CORTf or behavior compared to airworthy birds. As a result of the analysis, we found no significant differences in CORTf between flight-restricted and airworthy birds. However, reversibly deflighted pelicans had higher CORTf values than irreversibly deflighted and airworthy pelicans. In addition, pelicans living in groups consisting of more than 10 individuals showed lower CORTf values than pelicans in groups of less than 10 individuals. “Fluttering” behavior was significantly associated with higher CORTf values. In conclusion, the flight restriction of great white pelicans does not seem to impact the welfare indicators assessed in this study, adrenal activity, or behavior. The data show that the living conditions of pelicans (such as group size) may influence the welfare of these birds. To confirm this, further studies on other ground- and water-based birds are needed to provide more scientific data on animal welfare and living conditions in zoos.

**Abstract:**

The pinioning of birds was previously one of the most-accepted forms of mutilation in zoos. Despite a lack of knowledge on the effects of deflighting procedures with regard to the well-being of deflighted birds, pelicans are often reversibly deflighted by feather-clipping to keep them in open enclosures, including those with ponds without netting. In the present study, we focused on the welfare implications of flight restraint on one of the most commonly kept types of birds in German zoos, the great white pelican. A combination of behavioral observations and feather corticosterone concentrations (CORTf) of pelicans with different deflighting statuses (i.e., irreversibly deflighted, reversibly deflighted, and airworthy) was used to evaluate the effects of deflighting status on pelican welfare. We observed 215 individuals in 21 different German zoos. The pelicans lived in differently designed exhibits. An ethogram for these species was developed and their behavior was evaluated by scan sampling. Feather samples from 182 individuals were collected to determine if different deflighting conditions influenced the CORTf and therefore stress levels. The hypothesis was that the CORTf values of airworthy pelicans differ from those of deflighted pelicans. Tendencies with regard to the flight status groups were found. Conversely, reversibly deflighted pelicans had higher CORTf levels than irreversible deflighted and airworthy pelicans. Tendencies with regard to CORTf values and the group size of the kept pelicans were observed. The CORTf values were lower in groups consisting of more than 10 animals. In addition, the frequency of fluttering behavior was positively associated with CORTf values. Pelicans that frequently showed fluttering had higher CORTf values. Therefore, fluttering behavior might be considered a sign of stress levels in pelicans. This study is one of the first important steps in assessing the impact of deflighting procedures on the welfare of great white pelicans kept in zoos.

## 1. Introduction

The EC Zoos Directive (1992/22/EC) [1] states that animals must be accommodated under conditions that aim to satisfy the biological and conservational requirements of the individual species. A need remains to evaluate the welfare of zoo animals. Discussions about the well-being of zoologically kept animals have increased in the last decade [2,3]. 

All zoo animals deserve special attention. With regard to flying bird species, enclosure size and space to fly are two crucial aspects to consider when keeping fully winged birds in zoos. Often, large flying bird species under human care are prevented from escaping using a practice called pinioning. Although the debate is ongoing regarding this practice, to what extent pinioning affects the welfare of the animal and when the animal will adapt to the consequent physiological changes remain open questions [4,5,6,7,8]. Until now, scientists have not reached a consensus because of the lack of studies addressing this issue.

The legal regulations in these situations are diverse and vary from country to country, ranging from the prohibition of any deflighting procedures to their unequivocal permission [9]. In Germany, the Animal Welfare Law prohibits any type of surgical deflighting procedure, as regulated in Article 6 (1): The total or partial amputation of body parts or the complete or partial removal or destruction of organs or tissues of a vertebrate are prohibited, except where there is a veterinary indication. This law includes all vertebrates, meaning birds are not exempted [10].

In contrast, in Sweden, England, and Wales for example, the deflighting of zoo birds is allowed [11]. Reese et al. presented a detailed overview of the legal status of deflighting procedures in different countries [9].

In addition to these different legal regulations, there are various ethical views on the deflighting of zoo birds [9]. For example, it was argued that for pelicans, as water- and ground-based birds, the need to fly is not as important as the need for ponds and large terrestrial areas for walking [4].

Despite some difficulties, in the last decades efforts to improve and measure animal welfare have been made [12,13,14]. Animal welfare science has achieved important steps in developing valid animal welfare assessment tools, especially regarding the development and validation of animal-based indicators [15].

Within the zoo community, researchers incorporated glucocorticoid (GC) measurements to evaluate stress levels and thereby the welfare of their animals. Cortisol measurements are performed primarily by using the following matrices: blood, saliva, feces, and urine [16]. Although these methods are well-established and provide reliable results when used correctly, they only measure short-term circulating cortisol levels, over periods usually not exceeding 24 h. In recent years, because long-term GC measurements are useful for welfare purposes, other matrices such hair and feathers have been used for GC analyses. The use of these two matrices is based on GC being accumulated in the hair shaft and the feathers in direct proportion to its free concentration in the blood during matrix growth [17].

Bortolotti et al. [18] reported that measuring corticosterone (CORT) concentrations in feathers can be used as a non-invasive measure of integrative long-term GC levels. The authors showed that the feather CORT concentrations (CORTf) can be used as a measure of the hypothalamic–pituitary–adrenal (HPA) axis activity in red-legged partridge (*Alectoris rufa*). They found that CORT deposition in feathers correlates with plasma CORT levels. They also found that feathers, just like hair, provide a historical record of past HPA activity and, therefore represent a means to track stress over the time. Knowledge regarding the length of the developing feather, its growth rate, and the history of the bird regarding stressful situations enables the correlation of CORTf levels and stressful situations. In several studies, CORTf has been successfully measured to evaluate long-term stress in several species, from raptors to hens [19,20,21]. In zoo birds living under different flight restraint conditions, CORTf was measured in flamingos [22]. As pelicans are another popular bird species in zoos, the present study was conducted on the great white pelican.

In Europe, 129 zoos keep 1072 great white pelicans (*Pelecanus onocrotalus*) (Species 360, Zoological Information Management System (ZIMS)). In 24 German zoos, more than 260 great white pelicans are kept (ZIMS) under different conditions. These pelicans often live in large enclosures, including ponds without netting where flight restriction is needed.

Keeping airworthy pelicans that are among the largest flying birds in the world [23] necessitates a large free-flight aviary to provide the opportunity to fly. This is provided in a 1-hectare aviary for airworthy Dalmatian Pelicans (*Pelecanus crispus*) in ZooParc de Beauval in France. Another example of these large free-flight aviaries was constructed at Odense Zoo in Denmark in 2009. The mixed-species exhibit for African water birds (including pelicans, flamingos, spoonbills, and storks) was described by Klausen as an exciting interaction giving both keepers and visitors a new perspective on the life of these species [24]. Klausen argued that the birds appear to exhibit a full range of natural behaviors including flight, suggesting that the possibility of flight is an important aspect of ensuring the optimal welfare of great white pelicans under human care, because flying is part of the pelicans’ natural behavior [24].

The loss of flight restricts a range of behaviors, including the ability to escape, roost, and migrate [8]. Thus, Hestermann [8] discussed that deflighting can, in practice, be related to better opportunities to perform some other natural behaviors. Permanently deflighted birds can be kept in the open, which provides them more freedom to express natural behaviors such as foraging and exploring than conditions within the confines of an aviary.

In relation to welfare problems, Hestermann [8] underlined the importance of flying as response to escape from threats, especially predators. Thus, to ensure an optimal welfare level in deflighted birds, Hestermann stated that all free-range enclosures should be predator-safe. As such, the flight-restricted pelicans can display their natural behavior on secure grounds and be free from other stimuli that may provoke an antipredatory or escape response.

To date, no scientific studies have evaluated the effect of deflighting on great white pelicans under human care.

The contrasting regulations in many countries and the different individual opinions demonstrate the need for studies providing new knowledge on the question whether deflighted birds have a lower welfare status than airworthy ones as measured by stress indicators.

In this study, we investigated the impact of deflighting on great white pelicans kept in German zoological gardens using behavioral data and CORTf measurements to obtain animal-based data.

## 2. Materials and Methods

### 2.1. Ethics

This study was performed according to the guidelines of the German Animal Welfare Act and the European Directive 2010/63/EU for the protection of animals used for scientific purposes and was approved by the competent legal authorities of the respective German federal states where the participating zoos were located (approval number: 55.2 DMS 2532-2-337).

### 2.2. Study Design and List of the Participating Zoos

All German zoos keeping great white pelicans were asked to participate in this study (evaluated by Verband der Zootierärzte in 2015), with 22 pelican groups in the 21 out of 24 zoos agreeing to participate. Zoos provided the number, age, and sex of the animals, together with further information regarding animal husbandry such as breeding time and feeding procedures. The groups of great white pelicans were divided into 3 groups regarding their flight status: irreversibly deflighted (if they were pinioned or extirpated), reversibly deflighted (if they were feather-clipped), or airworthy (if they had intact wings and were potentially able to fly). The sample size calculation included the number of animals to be sampled to validate the study’s hypotheses with sufficient statistical power. To consider the expected cluster effect within the zoos, the number of animals per zoo was estimated based on preliminary data. Consequently, feathers had to be plucked from 10 randomly selected pelicans in each participating zoo irrespective of the defeathering status. If less than 10 individuals were kept, all animals were sampled. Concerning airworthy pelicans, all animals were sampled (see Table 1).

### 2.3. Behavioral Observation

Based on observations of a group of 9 great white pelicans at Vogelpark Marlow and considering Spot-Billed Pelicans (*Pelecanus philippensis*) from Gokula in 2012 [25] and pelicans kept at the zoo in Leipzig reported by Inge Meischner in 1958 [26], an ethogram was developed (Table 2). During this observation, 10 behavioral categories were defined, as described in Table 2.

This ethogram was applied to the behavioral observation of 215 pelicans in the participating zoos. Behavior was recorded by scan sampling twice a day for two hours on three consecutive days in a row at each zoo, for a total of 12 h observation per zoo. The behavioral observation occurred at the time before or after pelicans’ feeding time. Every zoo was visited between May and September 2016. The data were collected every 3 min on a tablet computer and analyzed using The Observer software from Noldus (The Observer XT, Wageningen, NL, USA). Life parameters were recorded for each pelican from zoo records.

### 2.4. Feather Collection

In winter 2016, feather collection started. In all zoos, feathers were collected individually, benefiting from routine capture due to animal relocation to winter quarters to avoid stress for the animals. For each animal, 5 to 10 feathers from the interscapular region were plucked to reach a sum length of 20 cm. All collected feathers were fully molted. They did not contain any blood; their growth was complete. The pulled feathers were stored in paper envelopes, dry at room temperature in a dark place, and after approximately 6 months, they were sent to the laboratory (Laboratorio de Análisis de Indicadores Hormonales, Estrés, Bienestar y Reproducción Animal (LAIHA)) of the Universitat Autònoma de Barcelona (UAB).

### 2.5. Corticosterone Extraction and Corticosterone Concentration Measurements

At the UAB, all feather samples were prepared and weighted as described by Reese et al. in 2020 [22]. A total feather length of 200 mm was needed for each pelican to homogenize feather samples between individuals. Therefore, 2–8 feathers of each feather sample were chosen to reach this length. The average length of the feather samples was 206 ± 32 mm.

Corticosterone was extracted using a methanol-based extraction technique based on Bortolotti [18], modified by Monclus [28], and described in detail by Reese et al. in 2020 [22].

The calamus of each single feather was cut off and each feather was weighed individually. With a ball mill (Retsch, MM 200 type), each feather sample was minced for 5 min with 25 Hz into feather particles of <2 mm. 1.5 mL methanol was added to each aliquot. The samples were vortexed (Vortex Mixer S0200-230 V-EU, Labnet International Inc., Edison, NJ, USA) for 30 min at room temperature; afterwards, they were incubated for 18 h at 37 °C (G24 Environmental Incubator Shaker, New Brunswick Scientific Co. Inc., Edison, NJ, USA). After incubation, the samples were centrifuged for 15 min at 6000 rpm. Then, 1 mL of the supernatant was placed into a new aliquot, and the samples were placed in an oven (Kendro Laboratory Products, Langenselbold, Germany) at 37 °C until total dryness. Subsequently, 0.25 mL of buffer solution was added to each sample, and shaken for another minute. After this, the samples were stored at −20 °C until analysis.

A Corticosterone ELISA Kit #402810 of the Neogen Corporation (Corticosterone ELISA kit, Neogen^®^ Corporation, Ayr, U.K.) was used. ELISA was performed according to the manufacturer’s instructions.

### 2.6. Statistics

Data of the behavioral observation and the corticosterone values were transferred to an MS Excel^®^ 2016 file and analyzed using IBM SPSS Statistics version 24. Continuous data were checked for normal distribution using descriptive statistics and visual inspection (histogram and Q-Q plot). Due to the skewed distribution, the corticosterone values were converted to logarithmic scales. The occurrence of behaviors is described in terms of percentages. The influence of life parameters, behavior, and deflighting status on the corticosterone values was analyzed by mixed linear regression models with zoos included as random factors. The group size was categorized into 1–5 birds, 6–10 birds, or >10 birds. Initially, each variable was analyzed individually to assess its influence on the logarithmic CORT levels. Based on these results, a multivariable mixed linear regression model was used to investigate the common influence factors.

A manual forward selection was performed based on the *p*-values of the univariable analysis. Variables were selected by the change of –2log likelihood after uptake of another variable into the model. The flight status was retained in this model because it was the key factor in the research study. Location was fitted as a random effect. Model diagnostics included visual inspection of normality and homoscedasticity of residuals.

The level of significance was set at *p* = 0.05.

## 3. Results

### 3.1. Behavioral Observations

**Figure 1:**
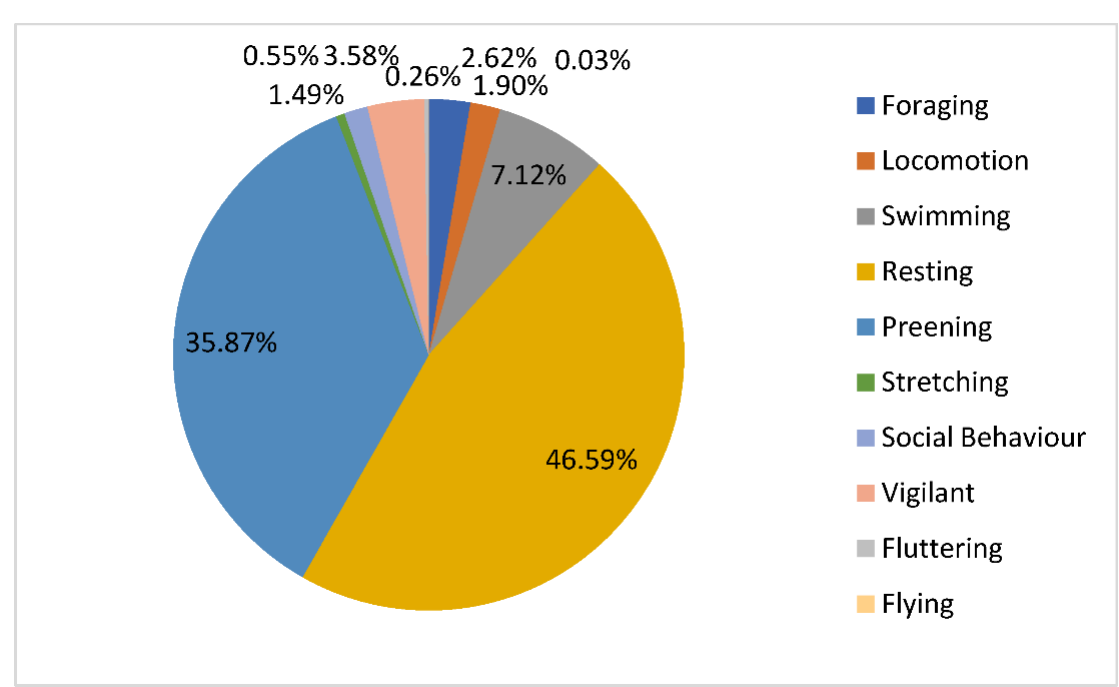
The average proportions of time spent by all 215 pelicans performing various behaviors.

The average frequency of the observed behavior for all pelicans is displayed in Figure 1.

Overall, more than 46% of the time the observed pelicans were resting. The proportion of time spent preening by the animals was 35.9%. These were the most frequently observed activities. The proportion of swimming was only 7% and the proportion of vigilant pelicans was 3.5%. Other observed activity proportions were <3.5%.

**Figure 2:**
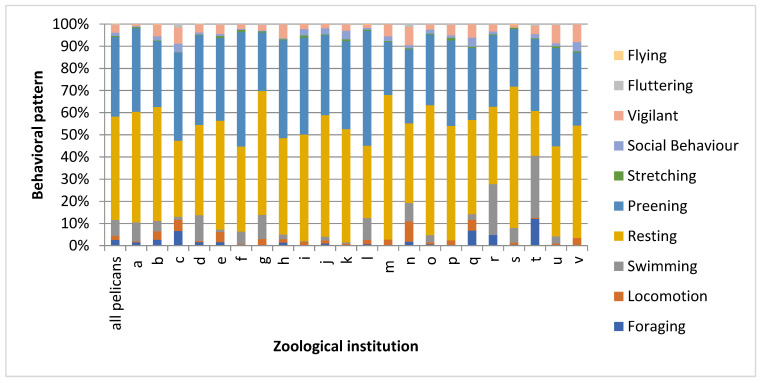
Activity of pelicans kept in 22 German zoos: the percentages of time that the animals spent on each behavior.

Figure 2 shows that the behavioral proportions differed between the zoos, but resting and preening were the two most frequently observed activities in each zoo. The proportion of swimming differed between 0% (two zoos) and 28%. One of the zoos showing no swimming behavior kept its pelicans in an aviary with a small pond and the other one without swimming behavior kept its pelicans in an aviary with a pond without fresh water delivery.

In zoos keeping airworthy pelicans, the proportion of flying was 0.09%.

### 3.2. Feather Corticosterone Concentrations

The CORTf levels showed tendencies between flight status, age, and group size of the pelicans. Feather-clipped pelicans had higher CORTf than pinioned, extirpated, and airworthy pelicans. Adult pelicans (>3 years) had lower CORTf values. CORTf values differed between group sizes: smaller groups had higher values.

The mean, minimum, and maximum CORTf values for all variables are displayed in Table 3.

Univariable analyses were carried out for each of the mentioned factors (Table 4). For the observational variables, only walking and fluttering had *p*-values <0.2. Size and location also had *p*-values <0.2. Thus, the final model included walking, fluttering, group size, age, sex, and deflighting status.

As the zoo was included as a random factor into the models, no *p*-value is presented for the differences between locations. For the final model, the percentage of variance attributed to the variance between the locations is provided.

In Table 4, all *p*-values of the univariable and multivariable model are presented.

In the final model, only one variable was significantly associated with the corticosterone values: fluttering (*p* = 0.043).

**Figure 3:**
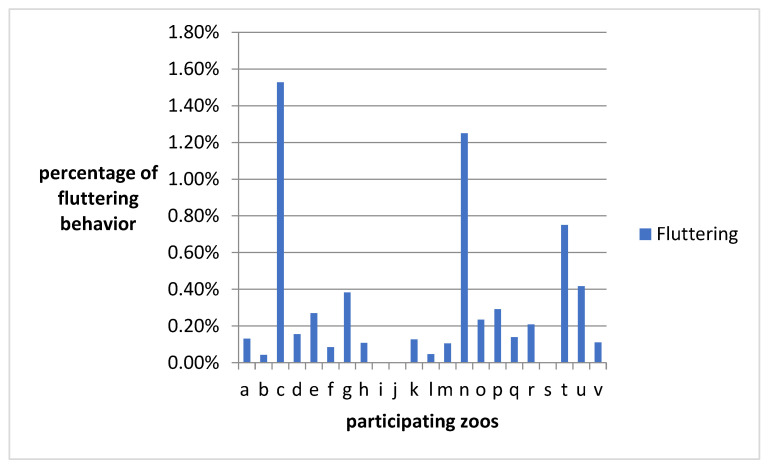
The percentage of fluttering at the different zoos.

Pelican groups in zoos with frequent fluttering had higher CORTf values. Figure 3 displays the percentage of fluttering at each zoo. The highest proportion of fluttering was found in zoos c and n; both zoos keep pelicans in groups with only three individuals.

## 4. Discussion

The findings of this study provide interesting insights into the associations between husbandry conditions, behavior, and CORTf levels in great white pelicans in German zoos. CORTf, as an animal-based welfare indicator, is able to provide some information about the stress experienced by the birds.

We obtained different findings through our investigation. We found no significant associations between CORTf values and the flight status per se; however, tendencies were detected as reversibly deflighted pelicans had higher CORTf values in comparison to irreversibly deflighted and airworthy pelicans.

The tendency towards higher CORTf values in reversibly deflighted pelicans may be explained by the predominately stressful procedure of feather-clipping in birds. The pelicans are captured at most twice per year to be feather-clipped. This procedure seems to be stressful for the captured pelicans and might be reflected in the higher CORTf values, as stressful situations can lead to increased CORTf [28]. This procedure occurs at different times of the year, mostly in spring and autumn. It could not be clearly verified if the plucked feathers of the pelicans included higher CORTf values due to the procedure of feather-clipping; nor can it be stated whether these elevated measurements reflect the HPA peak during the procedure or whether these are the result of generally higher HPA activity. Feather-clipped pelicans under human care might develop chronic stress caused by the fear of being caught again. In general, pelicans molt partially before the breeding season; complete molt is reached commonly during or after it [29]. Great white pelicans produce a colored plumage at the time of breeding [30]. This coloration provides evidence to determine the exact time of the breeding season of pelicans that mostly occurs in winter in German zoos. That would be comparable to the molt of brown pelicans (*Pelecanus occidentalis*), which molt mainly during their breeding season in winter [31]. Accordingly, interscapular feathers of the great white pelicans were plucked after a period of approximately 6 months regarding to the observation of pelicans and before the breeding season in winter.

The results of this study also reveal non-significant associations between CORTf, life parameters, and behavior. The finding that the pelicans’ sex did not affect CORTf is comparable to the findings of other studies such as those in northern bald ibises (*Geronticus eremita*) [32]. The age of the pelicans seems to affect CORTf. Adult pelicans (>3 years) showed lower CORTf values than juveniles. This is a similar to other findings in swallows (*Hirundo rustica*) [33].

Tendencies between CORTf and the group size of pelicans under human care were found. The group sizes ranged from 3 to 25 individuals. Groups with more than 10 individuals had lower CORTf concentrations than groups with less than 10 individuals. This is in line with findings in the wild. Great white pelicans breed in their natural habitat, for example, at the Danube Delta, in groups of thousands of pairs of pelicans [34]. The same applies to African colonies [31]. The reason for this may be antipredator strategies such as the many-eyes hypothesis or the dilution hypothesis [35], or it might be because of finding more and better food in these places [36]. Many advantages are obtained by animals living in larger groups [37]. Therefore, this finding is perhaps related to this natural behavior of pelicans, where pelicans living in groups with more than 10 individuals show lower CORTf. Another option is that zoos with larger groups of pelicans provide better husbandry than zoos with smaller groups. It is important to be cautious when drawing outcomes from the findings regarding the welfare of the great white pelican. The findings show tendencies, giving us only a direction toward the overall outcome of this study. Additional studies concentrating on these findings could verify these hypotheses.

A significant association between CORTf and fluttering behavior was found. As we performed a group behavioral assessment and only collected feather samples from some randomly selected individuals, we state that the connections are identified at the group level and not at the individual level. Groups of great white pelicans with higher frequency of fluttering behavior showed higher CORTf. This indicates that fluttering can possibly be an indicator for an increased allostatic load. At present, the reinforcers of this behavior are not known, nor is the evolutionary reason for fluttering clear. One possible explanation is as a flight or attack response or social stress or social conflict. Furthermore, it is unclear whether airworthy pelicans fly after fluttering. Individual behavioral assessments of each animal are necessary to find answers to these questions. Moreover, different factors need to be considered. For instance, further studies may focus on the size of the exhibit, the feature of the landscape, the size of the pond, interaction with visitors, interaction with animal keepers, interaction with other animals, feeding time, and feeding conditions. For water-based birds, the water quality of the pond and the size of the pond may other factors influencing pelicans’ behavior. In zoos where the ponds did not have the delivery of fresh water or the pond was small, the proportion of swimming behavior was 0%. All these conditions might impact different behaviors and CORTf values. The coherences between the aforementioned factors and behavior add valuable information regarding pelicans’ well-being in zoos. Fluttering, as a potential stress indicator, might be helpful in further research studies regarding the well-being of pelicans kept in zoos. Further studies should be performed to confirm these results. The finding of Voit et al. [38], that a non-invasive technique such as cutting feathers can be used instead of plucking feathers for CORTf measurement, may facilitate future studies. In flamingos, another ground- and water-based bird, no significance differences between flight status and CORTf values [22] were determined as in pelicans as well. Therefore, future studies should be conducted on other deflighted species at zoos to evaluate their welfare. For further studies it is important to include a balanced number of irreversibly deflighted, reversibly deflighted and airworthy pelicans to make an adequate comparison. Unfortunately, this was not possible in our study, as we could only include existing groups in the study. Further improvements to support the results include an extended time of behavioral observation and an individual behavioral assessment.

## 5. Conclusions

The results of this study demonstrate the necessity of the combination of behavioral observation and CORTf measurements to assess the well-being of zoo birds. This was a first promising approach for assessing the welfare of pelicans living in different zoos. The initial findings are informative and may offer the first approaches for the improvement of the pelicans’ well-being by considering for example fluttering behavior. The behavioral observation of pelicans and measurements of CORTf can only reveal part of the complex issue of animal welfare and need to be further investigated.

## Figures and Tables

**Table 1:** Overview of the participating zoos and their populations of great white pelicans and their flight status.

Zoo	Total Number of Observed Animals	Feather Samples for CORTf	Flight Status Airworthy	Flight Status Irreversibly Deflighted	Flight Status Reversibly Deflighted
a	18	10		10	
b	10	10		10	
c	3	3		3	
d	16	16		9	7
e	14	13		13	
f	5	5			5
g	25	10		10	
h	16	11		11	
i	8	8		8	
j	4	4		4	
k	10	10		6	4
l	9	9	1	8	
m	4	4	4		
n	4	4		4	
o	16	12		12	
p	10	10		6	4
q	3	3			3
r	8	8		4	4
s	9	9		7	
t	10	10	2	6	4
u	5	5		3	2
v	8	8			8
Total	215	182	7	134	41

**Table 2:** Ethogram of great white pelican.

Behavior Pattern	
Locomotion	Pelicans shift their weight from 1 side to another, 1 foot is set on the ground by the other in sequence. They roll over from the heel to the toes. The speed depends on the circumstances; they can also run. The wings are sometimes opened for balance.
Stretching	There are 2 types of stretching: the pelicans stretch their head horizontally while the wings are pulled backwards, or the pelicans stretch one foot horizontally backward and the equilateral wing is pulled backward.
Preening	There are different types of preening, but commonly, “preening involves the contact between the bill and feathers” [25]. The feathers of the whole body can be erected and laid straight back. The wings are opened during sunbathing to dry their feathers. Scratching is also a type of preening.
Swimming	Pelicans swim by paddling with their feet in the water.
Resting	They stand on their feet on the ground, or they lay down and their eyes can be closed or be open. The head is placed vertically on their back. In this position they often put their beak in the feathers on the back.
Foraging	Pelicans try to catch fish by diving with their head or with only their beak under water while opening their beak.
Social behavior	Social behavior is described as interactions between 2 or more pelicans. It can be antagonistic behavior or, for example, allopreening, when “it preens the plumage of another bird” [27].
Vigilant	If pelicans are alert, the neck will be stretched upward and the eyes will be open. Pelicans stand or sit in this position.
Fluttering	Fluttering is expressed by walking, running, or standing combined with moving of the wings.
Flying	If there is no connection to the ground, the pelicans are flying by moving their wings up and down.

**Table 3:** Mean, minimum, and maximum of logarithmic CORTf values in pg/mm; n = number of pelicans in the different zoos.

	n	Mean	Minimum	Maximum
**Status**				
Airworthy	7	1.53	1.24	1.95
Extirpated	6	1.46	1.05	1.73
Pinioned	128	1.53	0.89	2.45
Feather-clipped	41	1.67	1.17	2.25
**Group size**				
1–5 pelicans	28	1.71	1.18	2.45
6–10 pelicans	82	1.57	1.07	2.09
>10 pelicans	72	1.48	0.89	2.29
**Sex**				
Female	98	1.58	0.89	2.45
Male	84	1.54	1.12	2.29
**Age**				
<1 year	3	1.72	1.50	2.02
1–3 years	4	1.76	1.43	2.09
>3 years	175	1.55	0.89	2.45

**Table 4:** The *p*-values of the univariable and multivariable models.

Parameter	*p*-Value Univariable	Effect Size	*p*-Value Multivariable	Effect Size
Foraging	0.215	0.233		
Walking	0.081	0.283		
Swimming	0.611	0.103		
Resting	0.241	0.222		
Preening	0.757	0.058		
Stretching	0.688	0.079	0.260	0.197
Social behavior	0.296	0.188		
Vigilant	0.471	0.126		
Fluttering	0.030	0.303	0.043	0.282
Flying	0.284	0.218		
group size	0.154	001	0.083	0.1
Age	0.821	0.047/0.038	0.885	0.047/0.038
Sex			0.609	0.019
Status			0.946	0.021/0.020
